# Asthma

**DOI:** 10.1186/1710-1492-7-S1-S2

**Published:** 2011-11-10

**Authors:** Harold Kim, Jorge Mazza

**Affiliations:** 1 University of Western Ontario, London, Ontario, Canada; 2 McMaster University, Hamilton, Ontario, Canada; 3 Allergy Clinic, London Health Sciences Centre, London, Ontario, Canada

## Abstract

Asthma is the most common respiratory disorder in Canada. Despite significant improvement in the diagnosis and management of this disorder, the majority of Canadians with asthma remain poorly controlled. In most patients, however, control can be achieved through the use of avoidance measures and appropriate pharmacological interventions. Inhaled corticosteroids (ICSs) represent the standard of care for the majority of patients. Combination ICS/long-acting beta_2_-agonists (LABA) inhalers are preferred for most adults who fail to achieve control with ICS therapy. Allergen-specific immunotherapy represents a potentially disease-modifying therapy for many patients with asthma, but should only be prescribed by physicians with appropriate training in allergy. Regular monitoring of asthma control, adherence to therapy and inhaler technique are also essential components of asthma management. This article provides a review of current literature and guidelines for the appropriate diagnosis and management of asthma.

## Introduction

Asthma remains the most common chronic respiratory disease in Canada, affecting approximately 10% of the population [1]. Although asthma is often believed to be a disorder localized to the lungs, current evidence indicates that it may represent a component of systemic airway disease involving the entire respiratory tract, and this is supported by the fact that asthma frequently coexists with other atopic disorders, particularly allergic rhinitis [2].

Despite significant improvements in the diagnosis and management of asthma over the past decade, as well as the availability of comprehensive and widely-accepted national and international clinical practice guidelines for the disease, asthma control in Canada remains suboptimal. Results from the recent Reality of Asthma Control (TRAC) in Canada study suggest that over 50% of Canadians with asthma have uncontrolled disease [3]. Poor asthma control contributes to unnecessary morbidity, limitations to daily activities and impairments in overall quality of life [1].

This article provides an overview of diagnostic and therapeutic guideline recommendations from the Global Initiative for Asthma (GINA) and the Canadian Thoracic Society and as well as a review of current literature related to the pathophysiology, diagnosis, and appropriate treatment of asthma.

## Definition

Asthma is defined as a chronic inflammatory disease of the airways. The chronic inflammation is associated with airway hyperresponsiveness (an exaggerated airway-narrowing response to triggers, such as allergens and exercise), that leads to recurrent symptoms such as wheezing, dyspnea (shortness of breath), chest tightness and coughing. Symptom episodes are generally associated with widespread, but variable, airflow obstruction within the lungs that is usually reversible either spontaneously or with appropriate asthma treatment [4]**.**

## Pathophysiology

Asthma is associated with T helper cell type-2 (Th2) immune responses, which are typical of other atopic conditions. Various allergic (e.g., dust mites, cockroach residue, furred animals, moulds, pollens) and non-allergic (e.g., infections, tobacco smoke, cold air, exercise) triggers produce a cascade of immune-mediated events leading to chronic airway inflammation. Elevated levels of Th2 cells in the airways release specific cytokines, including interleukin (IL)-4, IL-5, IL-9 and IL-13, that promote eosinophilic inflammation and immunoglobulin E (IgE) production by mast cells. IgE production, in turn, triggers the release of inflammatory mediators, such as histamine and cysteinyl leukotrienes, that cause bronchospasm (contraction of the smooth muscle in the airways), edema (swelling) and increased mucous secretion (mucous hypersecretion), which lead to the characteristic symptoms of asthma [4,5].

The mediators and cytokines released during the early phase of an immune response to an inciting allergen, trigger a further inflammatory response (late-phase asthmatic response) that leads to further airway inflammation and bronchial hyperreactivity [5].

Evidence suggests that there may be a genetic predisposition for the development of asthma. A number of chromosomal regions associated with asthma susceptibility have been identified, such as those related to the production of IgE antibodies, expression of airway hyperresponsiveness, and the production of inflammatory mediators. However, further study is required to determine specific genes involved in asthma as well as the gene-environment interactions that may lead to expression of the disease [4,5].

## Diagnosis

The diagnosis of asthma involves a thorough medical history, physical examination, and objective assessments of lung function (spirometry preferred) to confirm the diagnosis (see Table 1). Bronchoprovocation challenge testing and assessing for markers of airway inflammation may also be helpful for diagnosing the disease, particularly when objective measurements of lung function are normal despite the presence of asthma symptoms [4,6,7].

**Table 1:** Diagnosis of asthma based on medical history, physical examination and objective measurements [4,6,7]

***Medical history*** • Assess for classic symptoms of asthma: – Wheezing – Breathlessness – Chest tightness – Cough (with our without sputum) • Assess for symptom patterns suggestive of asthma: – Recurrent/episodic – Occur/worsen at night or early in the morning – Occur/worsen upon exposure to allergens (e.g., animal dander, pollen, dust mites) or irritants (e.g., exercise, cold air, tobacco smoke, infections) – Respond to appropriate asthma therapy • Assess for family or personal history of atopic disease (particularly allergic rhinitis) ***Physical Examination*** • Examine for wheezing on auscultation • Examine upper respiratory tract and skin for signs of other atopic conditions ***Objective Measurements*** • Perform spirometry (preferred) to confirm the diagnosis – Diagnostic criteria: ■ FEV_1_ ↑ (after bronchodilator): ≥ 12% and ≥ 200 mL • Consider PEF as an alternative if spirometry is unavailable – Diagnostic criteria: ■ PEF ↑ (after bronchodilator): ≥ 20% and 60 L/min ■ Diurnal variation: >20% • If spirometry (or PEF) is normal, but symptoms are present consider: – Challenge testing (e.g., methacholine, histamine, mannitol, exercise) – Non-invasive markers of airway inflammation (exhaled nitric oxide, sputum eosinophilia) – Trial of appropriate asthma therapy ***Allergy testing*** • Perform skin tests to assess allergic status and identify possible triggers

FVC: forced vital capacity; FEV_1_: forced expiratory volume in 1 second; PEF: peak expiratory flow

### Medical history

The diagnosis of asthma should be suspected in patients with recurrent cough, wheeze, chest tightness and shortness of breath. Symptoms that are variable, occur upon exposure to allergens or irritants, that worsen at night, and that respond to appropriate asthma therapy are strongly suggestive of asthma [4,7]. Alternative causes of suspected asthma symptoms should be excluded, such as chronic obstructive pulmonary disease (COPD), bronchitis, chronic sinusitis, gastroesophageal reflux disease, recurrent respiratory infections, and heart disease.

A positive family history of asthma or other atopic diseases and/or a personal history of atopic disorders, particularly allergic rhinitis, can also be helpful in identifying patients with asthma. During the history, it is also important to examine for possible triggers of asthma symptoms, such as dust mites, cockroaches, animal dander, moulds, pollens, exercise, and exposure to tobacco smoke or cold air. Exposure to agents encountered in the work environment can also cause asthma. If work-related asthma is suspected, details of work exposures and improvements in asthma symptoms during holidays should be explored. It is also important to assess for comorbidities that can aggravate asthma symptoms, such as allergic rhinitis, sinusitis, obstructive sleep apnea and gastroesophageal reflux disease [7].

The diagnosis of asthma in young children is often more difficult since episodic wheezing and cough are common in this patient population and spirometry is unreliable in patients under 6 years of age. A useful method of confirming the diagnosis in young children is a trial of treatment with short-acting bronchodilators and inhaled corticosteroids (ICSs). Marked clinical improvement during treatment and deterioration upon cessation of therapy supports a diagnosis of asthma [4,8,9].

### Physical examination

Given the variability of asthma symptoms, the physical examination of patients with suspected asthma is often unremarkable. Physical findings are usually only evident if the patient is symptomatic. Therefore, the absence of physical findings does not rule out a diagnosis of asthma. The most common abnormal physical finding is wheezing on auscultation, which confirms the presence of airflow limitation [4]. Physicians should also examine the upper respiratory tract and skin for signs of concurrent atopic conditions such as allergic rhinitis or dermatitis [7].

### Objective measurements of lung function

Spirometry is the preferred objective measure to assess for reversible airway obstruction (i.e., rapid improvement in lung function after inhalation of a rapid-acting bronchodilator) and to confirm a diagnosis of asthma. It is recommended for all patients over 6 years of age who are able to undergo lung function testing [4,6].

Spirometry must be performed according to proper protocols. It is commonly performed in pulmonary function laboratories, but can also be performed in primary-care offices. During spirometry, the patient is instructed to take the deepest breath possible and then to exhale as hard and as fully as possible into the mouthpiece of the spirometer.

Spirometry measures the forced vital capacity (FVC, the maximum volume of air that can be exhaled) and the forced expiratory volume in 1 second (FEV_1_). The ratio of FEV_1_ to FVC provides a measure of airflow obstruction. A diagnosis of asthma is confirmed when there is: (1) an improvement in FEV_1_ of at least 12% and at least 200 mL 15–20 minutes after administration of an inhaled rapid-acting bronchodilator, or (2) an improvement in FEV_1_ of at least 20% and at least 200 mL after 2 weeks of treatment with an anti-inflammatory agent. In the general population, the FEV_1_/FVC ratio is usually greater than 0.80 (and possibly greater than 0.90 in children) and, therefore, any values less than these suggest airflow limitation and also support a diagnosis of asthma. Because of the variability of asthma symptoms, patients will not exhibit reversible airway obstruction at every visit. Therefore, to increase sensitivity, spirometry should be repeated, particularly when patients are symptomatic [6,7].

Peak expiratory flow (PEF) monitoring is an acceptable alternative when spirometry is not available, and can also be useful for diagnosing occupational asthma and/or monitoring response to asthma treatments. PEF is usually measured in the morning and in the evening. A diurnal variation in PEF of more than 20% or an improvement of at least 60 L/min or at least 20% after inhalation of a rapid-acting bronchodilator suggests asthma [6]. Although simpler to perform than spirometry, PEF is not as reliable. Therefore, as mentioned earlier, spirometry is the preferred method of documenting airflow limitation and confirming the diagnosis of asthma.

### Challenge testing

When lung function tests are normal, but symptoms suggest asthma, measurements of airway responsiveness using direct airway challenges to inhaled bronchoconstrictor stimuli (e.g., methacholine or histamine) or indirect challenges with mannitol or exercise may help confirm a diagnosis of asthma.

Challenge testing should be conducted in accordance with strict protocols in a laboratory or other facility equipped to manage acute bronchospasms. Testing involves the patient inhaling increasing doses or concentrations of a stimulus until a given level of bronchoconstriction is achieved, typically a 20% fall in FEV_1_. An inhaled rapid-acting bronchodilator is then provided to reverse the obstruction. Test results are usually expressed as the dose or concentration of the provoking agent that causes the FEV_1_ to drop by 20% (the PD_20_ or PC_20_, respectively). For methacholine, a PC_20_ value less than 8 mg/mL is considered a positive result indicative of airway hyperreactivity, and supports a diagnosis of asthma. However, positive challenge tests are not specific to asthma and may occur with other conditions such as allergic rhinitis and COPD. Therefore, challenge testing may be most useful for ruling out asthma. A negative test result in a symptomatic patient not receiving anti-inflammatory therapy is highly sensitive for ruling out the disease [7].

Challenge testing is contraindicated in patients with FEV_1_ values less than 60-70% of the normal predicted value (since bronchoprovocation could cause significant bronchospasms), in patients with uncontrolled hypertension or in those who recently experienced a stroke or myocardial infarction [10].

### Non-invasive markers of airway inflammation

The measurement of inflammatory markers such as sputum eosinophilia (amount of eosinophils in the sputum) or levels of exhaled nitric oxide (a gaseous molecule produced by some cells during an inflammatory response) can also be useful for diagnosing asthma. Evidence suggests that exhaled nitric oxide levels may be better able to identify asthmatic patients than basic lung function testing, and may also be useful for monitoring patient response to asthma therapy [7]. Although these tests have been studied in the diagnosis and monitoring of asthma, they are not yet widely used in Canada. With further clinical evidence and use, these markers of airway inflammation will likely become more commonly available.

### Allergy skin testing

Allergy skin testing is also recommended to determine the allergic status of the patient and to identify possible asthma triggers. Testing is typically performed using the allergens relevant to the patient’s geographic region. Although allergen-specific IgE tests that provide an *in vitro* measure of a patient’s specific IgE levels against particular allergens have been suggested as an alternative to skin tests, these tests are less sensitive and more expensive than skin tests [4,6].

## Treatment

**Figure 1:**
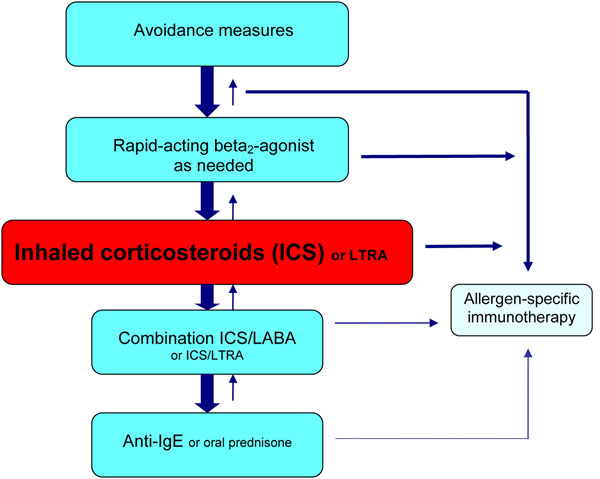
**A simplified, stepwise algorithm for the treatment of asthma. ***ICS: inhaled corticosteroid; LTRA: leukotriene receptor antagonist; LABA: long-acting beta_2_-agonist; IgE: immunoglobulin E*** Note: Treatments can be used individually or in any combination.**

The primary goal of asthma management is to achieve and maintain control of the disease in order to prevent exacerbations (abrupt and/or progressive worsening of asthma symptoms that often require immediate medical attention and/or the use of oral steroid therapy) and reduce the risk of morbidity and mortality. The level of asthma control should be assessed at each visit using the criteria in Table 2, and treatment should be tailored to achieve control. In most asthma patients, control can be achieved through the use of both avoidance measures and pharmacological interventions. The pharmacologic agents commonly used for the treatment of asthma can be classified as controllers (medications taken daily on a long-term basis that achieve control primarily through anti-inflammatory effects) and relievers (medications used on an as-needed basis for quick relief of bronchoconstriction and symptoms). Controller medications include ICSs, leukotriene receptor antagonists (LTRAs), long-acting beta_2_-agonists (LABAs) in combination with an ICS, and anti-IgE therapy. Reliever medications include rapid-acting inhaled beta_2_-agonists and inhaled anticholinergics [4,6,7]. Allergen-specific immunotherapy may also be considered in most patients with allergic asthma, but must be prescribed by physicians who are adequately trained in the treatment of allergies [11,12]. Systemic corticosteroid therapy may also be required for the management of acute asthma exacerbations. A simplified, stepwise algorithm for the treatment of asthma is provided in Figure 1.

**Table 2:** Criteria for assessing asthma control [4,6]

• No exacerbations • Fewer than 3 doses per week of a rapid-acting beta_2_-agonist bronchodilator • Daytime symptoms < 3 days per week • No nighttime symptoms • Normal physical activity • No absenteeism from work or school • FEV_1_ or PEF at least 90% of personal best

FEV_1_: forced expiratory volume in 1 second; PEF: peak expiratory flow

When asthma control has been achieved, ongoing monitoring is essential to establish the minimum maintenance doses required to maintain control. However, because asthma is a variable disease, treatment may need to be adjusted periodically in response to loss of control (as indicated by failure to meet the control criteria in Table 2) [4]. It is also imperative that all asthma patients be empowered to take an active role in the management of their disease. This can be accomplished by providing patients with a personalized written action plan for disease management and by educating the patient about the nature of the disease, the role of medications, the importance of adhering to controller therapy, and the appropriate use of inhaler devices [7].

### Avoidance measures

Avoidance of relevant allergens/irritants is an important component of asthma management. Patients allergic to house dust mites should be instructed to use allergen-impermeable covers for bedding and to keep the relative humidity in the home below 50% (to inhibit mite growth). Pollen exposure can be reduced by keeping windows closed, using an air conditioner, and limiting the amount of time spent outdoors during peak pollen seasons. For patients allergic to animal dander, removal of the animal from the home is recommended and usually results in a significant reduction in symptoms within 4-6 months. However, compliance with this recommendation is poor and, therefore, the use of high-efficiency particulate air (HEPA) filters and restricting the animal from the bedroom or to the outdoors may be needed to attempt to decrease allergen levels. Measures for reducing exposure to mould allergens include cleaning with fungicides, de-humidification to less than 50%, and HEPA filtration. Cigarette smoking and exposure to second-hand tobacco smoke should also be avoided [7].

Since these avoidance strategies can be labour-intensive, patient adherence is usually suboptimal. Frequent reassessments, encouragement and empowerment by the treating physician are often required to help promote adherence to these strategies. Furthermore, patients should be advised to use a combination of avoidance measures for optimal results, since single-strategy interventions have demonstrated no measurable benefits in asthma control [7].

### Reliever medications

Inhaled rapid-acting beta_2_-agonists are the preferred reliever medications for the treatment of acute symptoms, and should be prescribed to all patients with asthma. In Canada, several short-acting beta_2_-agonists (SABAs; e.g., salbutamol, terbutaline) and one LABA (formoterol) are approved for this indication. SABAs should only be taken on an as needed basis for symptom relief. Increased use (i.e., 3 or more times per week) indicates worsening control and signals the need to reassess treatment to achieve control of symptoms. Unlike other LABAs, formoterol has a rapid onset of action and, therefore, can be used for acute symptom relief. However, given that LABA monotherapy has been associated with an increased risk of asthma-related morbidity and mortality, formoterol should only be used as a reliever in patients 12 years of age or older who are on regular controller therapy with an ICS [4,6,7].

Short-acting anticholinergic bronchodilators, such as ipratropium bromide, may also be used as reliever therapy. However, these agents appear to be less effective than inhaled rapid-acting beta_2_-agonists and, therefore, should be reserved as second-line therapy for patients who are unable to use SABAs. They may also be used in addition to SABAs in patients experiencing moderate to severe asthma exacerbations. Furthermore, chronic, short-acting anticholinergic bronchodilator therapy is not recommended for use in children [6].

### Controller medications

#### Inhaled corticosteroids (ICSs)

ICSs are the most effective anti-inflammatory medications available for the treatment of asthma and represent the mainstay of therapy for most patients with the disease. Low-dose ICS monotherapy is recommended as first-line maintenance therapy for most children and adults with asthma. Regular ICS use has been shown to reduce symptoms and exacerbations, and improve lung function and quality of life. However, ICSs do not “cure” asthma, and symptoms tend to recur within weeks to months of ICS discontinuation. Therefore, most patients will require long-term, if not life-long, ICS treatment [4,6,7].

Since ICSs are highly effective when used optimally, factors other than treatment efficacy need to be considered if ICS therapy is unsuccessful in achieving asthma control. These factors include: misdiagnosis of the disease, poor adherence to ICS therapy, improper inhaler technique, or the presence of other comorbidities. If, after addressing such factors, patients fail to achieve control with low-to-moderate ICS doses, then treatment should be modified. For most children, ICS dose escalation (to a moderate dose) is the preferred approach to achieve control, while the addition of another class of medications (usually a LABA) is recommended for patients over 12 years of age [6,9].

The most common local adverse events associated with ICS therapy are oropharyngeal candidiasis (also known as oral thrush) and dysphonia (hoarseness, difficulty speaking). Rinsing and expectorating (spitting) after each inhalation and/or the use of a spacer device can help reduce the risk of these side effects. Systemic adverse effects with ICS therapy are rare, but may include adrenal suppression, changes in bone density, cataracts, glaucoma and growth retardation [4].

#### Leukotriene receptor antagonists (LTRAs)

The LTRAs montelukast and zafirlukast are also effective for the treatment of asthma and are generally considered to be safe and well tolerated. However, because these agents are less effective than ICS treatment when used as monotherapy, they are usually reserved for patients who are unwilling or unable to use ICSs. LTRAs can also be used as add-on therapy if asthma is uncontrolled despite the use of low-to-moderate dose ICS therapy. It is important to note, however, that LTRAs are considered to be less effective than LABAs as add-on therapy in adults [4,6]. In children, however, the data are less clear and, therefore, the child’s symptoms and the presence of comorbidities may help direct treatment. For example, if a child with asthma also has allergic rhinitis, the addition of montelukast should be considered. If, however, the child has persistent airway obstruction, the addition of a LABA may be preferred.

#### Combination ICS/LABA inhalers

As mentioned earlier, LABA monotherapy is not recommended in patients with asthma as it does not impact airway inflammation and is associated with an increased risk of morbidity and mortality. LABAs are only recommended when used in combination with ICS therapy. The combination of a LABA and ICS has been shown to be highly effective in reducing asthma symptoms and exacerbations, and is the preferred treatment option in adolescents or adults whose asthma is inadequately controlled on low-dose ICS therapy, or in children over 6 years of age who are uncontrolled on moderate ICS doses [6]. Although there is no apparent difference in efficacy between ICSs and LABAs given in the same or in separate inhalers, combination ICS/LABA inhalers are preferred because they preclude use of the LABA without an ICS, are more convenient and may enhance patient adherence. Three combination ICS/LABA inhalers are available in Canada: salmeterol/fluticasone (Advair), budesonide/formoterol (Symbicort) and mometasone/formoterol (Zenhale). Combination budesonide/formoterol has recently been approved for use in Canada as a single inhaler for both daily maintenance (controller) and reliever therapy in individuals 12 years of age and older. It should only be used in patients whose asthma is not adequately controlled with low-to-moderate ICS doses or whose disease severity warrants treatment with combination therapy [4,6].

#### Theophylline

Theophylline is an oral bronchodilator with modest anti-inflammatory effects. Given its narrow therapeutic window and frequent adverse events (e.g., gastrointestinal symptoms, loose stools, seizures, cardiac arrhythmias, nausea and vomiting), its use is generally reserved for patients whose asthma is uncontrolled despite an adequate trial of ICS, LABAs and/or LTRAs [4,6].

#### Anti-IgE therapy

The anti-IgE monoclonal antibody, omalizumab, has been shown to reduce the frequency of asthma exacerbations by approximately 50%. The drug is administered subcutaneously once every 2-4 weeks and is approved in Canada for the treatment of moderate to severe, persistent allergic asthma in patients 12 years of age or older. At present, omalizumab is reserved for patients with difficult to control asthma who have documented allergies and whose asthma symptoms remain uncontrolled despite ICS therapy [6].

Table 3 provides a list of the commonly used controller therapies and their recommended dosing regimens. It is important to note that long-term compliance with controller therapy is poor because patients tend to stop therapy when their symptoms subside. Therefore, regular follow-up visits are important to help promote treatment adherence.

**Table 3:** Overview of the main controller therapies used for the treatment of asthma.

	Usual Adult Dose	Usual Pediatric Dose
**ICSs**		

Beclomethasone (Qvar, generics)	MDI: 100–800 µg/day, divided bid	MDI: 100-200 μg/day, divided bid (for children 5-11 years)

Budesonide (Pulmicort)	DPI: 400–2400 µg/day, divided bid Nebules: 1–2 mg bid	DPI: 200-400 μg/day, divided bid (not indicated for children < 6 years) Nebules: 0.25-0.5 mg bid (for children 3 months to 12 years)

Ciclesonide (Alvesco)	MDI: 100–800 µg/day	MDI: 100–200 µg/day (not indicated for children < 6 years)

Fluticasone (Flovent HFA, Flovent Diskus)	MDI/DPI: 100–500 µg bid	MDI/DPI: 50–200 µg bid (for children 4-16 years) MDI: 100 µg bid (via a pediatric spacer device with a face mask) (for children 12 months to 4 years)

**Combination ICS/LABA inhalers**		

Budesonide/formoterol (Symbicort)	DPI (maintenance): 100/6 µg or 200/6 µg, 1–2 puffs od or bid; max 4 puffs/day DPI (maintenance and reliever): 100/6 µg or 200/6 µg, 1–2 puffs bid or 2 puffs od; plus 1 puff as needed for relief of symptoms (no more than 6 puffs on any single occasion); max 8 puffs/day	Not indicated for children under 12 years of age

Fluticasone/salmeterol (Advair MDI, Advair Diskus)	MDI: 125/25 µg or 250/25 µg, 2 puffs bid Diskus: 100/50 µg, 250/50 µg or 500/50 µg: 1 puff bid	MDI: not indicated for children under 12 years of age Diskus: 100/50 µg, 1 puff bid (not indicated for children <4 years)

Mometasone/ formoterol (Zenhale)	For patients previously treated with: – Low-dose ICS: 50/5 µg, 2 puffs bid – Medium-dose ICS: 100/5 µg, 2 puffs bid – High-dose ICS: 200/5 µg, 2 puffs bid	Not indicated for children under 12 years of age

**LTRAs**		

Montelukast (Singulair)	10 mg tablet od (taken in the evenings)	5 mg chewable tablet od (taken in the evenings) (for children 6-14 years) 4 mg chewable tablet od or 4 mg packet of granules od (taken in the evenings) (for children 2-5 years of age)

Zafirlukast (Accolate)	20 mg tablet bid, at least 1 h before or 2 h after meals	Not indicated for children under 12 years of age

**Anti-IgE therapy**		

Omalizumab (Xolair)	150–375 mg sc every 2–4 weeks (based on patient’s weight and pre-treatment serum IgE level)	Not indicated for children under 12 years of age

ICS: inhaled corticosteroid; MDI: metered dose inhaler; DPI: dry powder inhaler; LTRA: leukotriene receptor antagonists; IgE: immunoglobulin E; od: once daily; bid: twice daily; sc: subcutaneously

#### Systemic corticosteroids

Systemic corticosteroids, such as oral prednisone, are generally used for the acute treatment of moderate to severe asthma exacerbations. While chronic systemic corticosteroid therapy may also be effective for the management of difficult to control asthma, prolonged use of oral steroids are associated with well-known and potentially serious adverse effects and, therefore, their long-term use should be avoided if at all possible. Adverse events with short-term, high-dose oral prednisone are uncommon, but may include: reversible abnormalities in glucose metabolism, increased appetite, edema, weight gain, rounding of the face, mood alterations, hypertension, peptic ulcers and avascular necrosis [4].

### Allergen-specific immunotherapy

Allergen-specific immunotherapy involves the subcutaneous administration of gradually increasing quantities of the patient’s relevant allergens until a dose is reached that is effective in inducing immunologic tolerance to the allergen. Although it has been widely used to treat allergic asthma, it is not universally accepted by all clinical practice guideline committees due to the potential for serious anaphylactic reactions with this form of therapy [11].

A Cochrane review of 75 randomized controlled trials examining the use of allergen-specific immunotherapy in asthma management confirmed its efficacy in reducing asthma symptom scores and medication requirements, and improving airway hyperresponsiveness [12]. Similar benefits have been noted with sublingual immunotherapy [13], which is expected to be approved in Canada in the near future. Evidence also suggests that allergen-specific immunotherapy may prevent the onset of asthma in atopic individuals [14].

At present, allergen-specific immunotherapy should be considered on a case-by-case basis. It can be used prior to a trial of ICS therapy in patients with very mild allergic asthma and concomitant allergic rhinitis and as add-on therapy in patients using ICSs alone [15]. Allergen-specific immunotherapy may also be considered in patients using combination inhalers, ICS/LTRAs and/or omalizumab if asthma symptoms are controlled. Since allergen-specific immunotherapy carries the risk of anaphylactic reactions, it should only be prescribed by physicians who are adequately trained in the treatment of allergy. The injections must be given in clinics that are equipped to manage possible life-threatening anaphylaxis where a physician is present. Furthermore, to reduce the risk of serious reactions, asthma must be controlled and the FEV_1_ > 70% of predicted at the time of each immunotherapy injection.

## Conclusions

Asthma is the most common respiratory disorder in Canada, and contributes to significant morbidity and mortality. A diagnosis of asthma should be suspected in patients with recurrent cough, wheeze, chest tightness and dyspnea, and should be confirmed using objective measures of lung function (spirometry preferred). Allergy testing is also recommended to identify possible triggers of asthma symptoms.

In most patients, asthma control can be achieved through the use of avoidance measures and appropriate pharmacological interventions. ICSs represent the standard of care for the majority of asthma patients. For those who fail to achieve control with low-to-moderate ICS doses, combination therapy with a LABA and ICS is the preferred treatment choice in most adults. LTRAs can also be used as add-on therapy if asthma is uncontrolled despite the use of low-to-moderate dose ICS therapy, particularly in patients with concurrent allergic rhinitis. Anti-IgE therapy may be useful in select cases of difficult to control asthma. Allergen-specific immunotherapy is a potentially disease-modifying therapy, but should only be prescribed by physicians with appropriate training in allergy. All patients with asthma should have regular follow-up visits during which criteria for asthma control, adherence to therapy and proper inhaler technique should be reviewed.

## Key take-home messages

• A clinical diagnosis of asthma should be suspected in patients with intermittent symptoms of wheezing, coughing, chest tightness and breathlessness.

• Objective measurements of lung function, preferably using spirometry, are needed to confirm the diagnosis.

• All asthma patients should be prescribed a rapid-acting bronchodilator to be used as needed for relief of acute symptoms.

• ICS therapy is the standard of care for most patients with asthma.

• Combination ICS/LABA inhalers are recommended for most adult patients who fail to achieve control with low-to-moderate ICS doses.

• LTRAs can also be used as add-on therapy if asthma is uncontrolled despite the use of low-to-moderate ICS doses.

• Allergen-specific immunotherapy is a potentially disease-modifying therapy that can be considered in most cases of allergic asthma.

• Regular monitoring of asthma control, adherence to therapy and inhaler technique are important components of asthma management.

## Competing interests

Dr. Harold Kim is the past president of the Canadian Network for Respiratory Care and co-chief editor of *Allergy*, *Asthma and Clinical Immunology*. He has received consulting fees and honoraria for continuing education from AstraZeneca, GlaxoSmithKline, Graceway Pharmaceuticals, King Pharma, Merck Frosst, Novartis, and Nycomed.

Dr. Jorge Mazza has received consulting fees and honoraria from Astra-Zeneca, GlaxoSmithKline, Graceway Pharmaceuticals and Novartis.
